# Evaluation of Children and Adolescents with Thalassemia Major in Terms of Osteoporosis: A Single-Centre Experience

**DOI:** 10.3390/jcm14051579

**Published:** 2025-02-26

**Authors:** Özhan Orhan, Hasan Demir, Mehmet Nur Talay, Nezir Özgün, Mehmet Nuri Özbek

**Affiliations:** 1 Department of Pediatrics, Faculty of Medicine, Mardin Artuklu University, Mardin 47100, Turkey; demir.hasan3447@gmail.com (H.D.); mntalay70@gmail.com (M.N.T.); 2 Department of Pediatric Neurology, Faculty of Medicine, Mardin Artuklu University, Mardin 47100, Turkey; nezirozgun@hotmail.com; 3 Department of Pediatric Endocrinology, Faculty of Medicine, Mardin Artuklu University, Mardin 47100, Turkey; drmnozbek@hotmail.com

**Keywords:** thalassemia major, child, bone mineral density, ferritin, calcium

## Abstract

**Background/Objectives**: This study aimed to determine the frequency of osteoporosis in children and adolescents with thalassemia major (TM) and to identify risk factors for the early development of osteoporosis. **Methods**: This retrospective study included 27 patients under 18 years of age receiving regular blood transfusions and chelation therapy for TM at our hospital. Bone mineral density (BMD) was measured by dual-energy x-ray absorptiometry, and a lumbar spine Z-score <−2 was considered osteoporotic. Patients with osteoporosis were classified as Group 1 and those without osteoporosis as Group 2. **Results:** Osteoporosis was detected in 22.2% of the study population. The mean age was 13.83 ± 2.85 years in Group 1 and 7.95 ± 5.05 years in Group 2 (*p* = 0.012). Body weight and height were significantly lower in Group 1 (*p* = 0.012 and *p* = 0.004). Ferritin levels were 5306 ± 1506 ng/mL in Group 1 and 2020 ± 1205 ng/mL in Group 2, and the difference was significant (*p* = 0.001). Group 1 had significantly lower Ca and P levels (*p* < 0.001, *p* = 0.038). BMD was negatively correlated with ferritin (r = −0.791, *p* < 0.001) and positively correlated with calcium (r = 0.499, *p* = 0.008). **Conclusions**: Osteoporosis is a common condition in TM patients. Patients with risk factors should be followed more closely. These patients should be identified before BMD decreases. To prevent osteoporosis, regular BMD scans should be performed, calcium and vitamin D supplementation should be provided, and physical activity should be encouraged.

## 1. Introduction

Thalassemias are genetic anemias that result from mutations affecting the process of hemoglobin synthesis. In Turkey, thalassemia has a prevalence of 0.37–0.6% and is largely seen in the southern part of the country. Over the last decade, prevention programmes implemented in Turkey have reduced the birth rate of babies with haemoglobinopathy by 90% [1,2,3].

Thalassemia major (TM) is the most severe form of the thalassemia disease. The symptoms of TM usually appear in the first years of life. Severe anaemia and hepatosplenomegaly are often present, but a wide variety of symptoms may be present, including failure to thrive, susceptibility to infection, fever, vomiting, diarrhoea, and pallor. Endocrinological dysfunction may also occur in patients with TM, and they may develop growth retardation, hypoparathyroidism, hypothyroidism, delayed puberty, diabetes mellitus, or adrenal failure [4,5].

Patients with TM also suffer bone deformities such as the enlargement of the cranial and facial bones, rickets, and spinal deformities. Furthermore, osteoporosis may also occur in these patients [3,6,7]. Many factors including reduced insulin-like growth factor levels, iron overload, cortical thinning of bones, and the negative effects of chelators administered to treat the disease on calcium (Ca) and phosphorus (P) uptake can lead to osteoporosis in these patients [7,8,9].

Early detection of osteoporosis in TM patients has an important role in preventing the unwanted effects of osteoporosis, such as skeletal abnormalities, fractures, vertebra deformities, nerve compression, and growth retardation [8,9].

There are no definitive data on the frequency of osteoporosis and the factors affecting patients with TM, meaning there is a need for more research. Thus, the primary aim of this study was to determine the frequency of osteoporosis and its relationship with biochemical, demographic, and clinical parameters in children and adolescents with TM followed at our clinic. Our secondary aim was to identify osteoporosis risk factors in patients with TM, which will allow early detection of at-risk groups.

## 2. Materials and Methods

**Figure 1:**
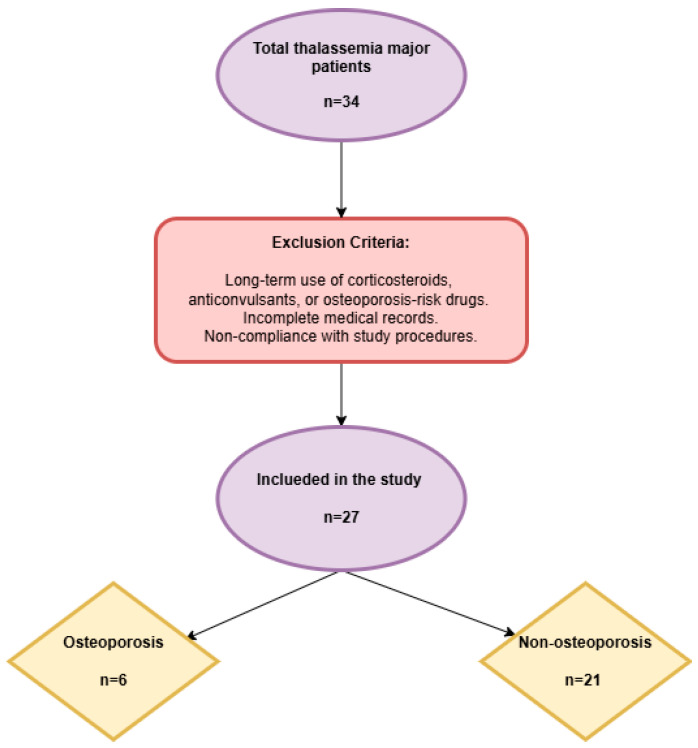
Study flowchart.

This retrospective study included patients under 18 years of age who were diagnosed with TM and received regular blood transfusion and chelator therapy at our tertiary care hospital between 1 January and 31 December 2024. Patients with long-term use of corticosteroids, anticonvulsants, or medications increasing the risk of osteoporosis; those with incomplete medical records; and those who failed to comply with the study procedures were excluded (Figure 1).

Data on the baseline demographic characteristics of the patients such as age, sex, transfusion frequency, height, weight, chelators, and medications were noted using medical records. Their height and weight were measured and body mass index (BMI) calculated. Ca, P, alkaline phosphatase (ALP), vitamin 25 hydroxy (OH) D, parathyroid hormone (PTH), and ferritin levels were measured from the blood samples collected before transfusion. Bone mineral density (BMD) was measured from the lumbar vertebrae using the dual-energy X-ray absorptiometry (DXA) method (Osteosys brand, Primus model), and the BMD z-score was adjusted for height. Patients with a DXA z-score of <−2 and those found to have microfractures were considered to have osteoporosis [10]. Patients with osteoporosis were classified as Group 1 and those without osteoporosis as Group 2.

In patients with TM, ineffective erythropoiesis causes enlargement of the bone marrow cavity, resulting in a decrease in cortical and trabecular bone tissue. The lumbar spine is thought to be more affected because it is primarily composed of trabecular bone and has a large bone marrow cavity [11]. The diagnosis of osteoporosis in children and adolescents should not be based solely on densitometric criteria. To minimise the influence of bone size on BMD measurements in the growing skeleton in studies of patients in this group, the apparent volumetric density of the lumbar spine was calculated using a specific formula according to age and sex [12,13,14]. In our study, the lumbar vertebral (L1–4) BMD results of the Turkish Paediatric Endocrinology and Diabetes Association were calculated according to age and gender with the help of automatic software [15].

The study was approved by the Mardin Artuklu University Ethics Committee for Noninvasive Clinical Research (No: 2024/4–16, date: 16 April 2023).

### Statistical Analysis

Statistical analyses were performed using IBM SPSS Statistics software (version 26.0, Chicago, IL, USA). The relationship between categorical variables and groups was analyzed using the Chi-square or Fisher’s exact test. The differences between the means of continuous variables in the two independent groups were analyzed using an ındependent samples *t*-test, and the results were reported as mean ±SD. The normality of study data was analyzed using the Shapiro–Wilk test. The Pearson correlation test was used to analyze the correlations between the study data. Univariate and multivariate logistic regression analyses were performed between two groups of children with and without osteoporosis. Variables with *p* < 0.05 in the univariate model were included as covariates in the multivariate model. All values were presented as odds ratios (ORs) and 95% confidence intervals (CIs) in univariate and multivariate logistic regression models. *p* values of less than 0.05 were considered statistically significant.

## 3. Results

A total of 27 patients diagnosed with TM were enrolled. The mean age was 13.83 ± 2.85 years in Group 1 and 7.95 ± 5.05 years in Group 2 (*p* = 0.012). There was no significant difference between the groups with respect to gender distribution (*p* = 0.675). Body weight and height were significantly lower in Group 1 (*p* = 0.012 and *p* = 0.004, respectively). A total of 5 patients had short stature, and 3 patients were underweight in Group 1. One patient was in the prepubertal period, while two patients had puberte tarda. No significant difference was found between the groups with respect to chelator dose and transfusion frequency. However, patients in Group 1 had a higher ferritin level. The splenic size was significantly higher in Group 1 (189.17 ± 6.43 mm) than in Group 2 (126.48 ± 37.95 mm) (*p* = 0.001). The demographic and clinical data of the patients are shown in Table 1.

The biochemical values of the patients are shown in Table 2. Group 1 had significantly lower Ca and *p* levels (*p* < 0.001, *p* = 0.038, respectively). Although ALP and PTH levels were higher, and vitamin 25 (OH) D levels were lower, and there was no statistical significance. Ferritin levels were significantly higher in Group 1 (5306 ± 1506 ng/mL) than in Group 2 (2020 ± 1205 ng/mL) (*p* = 0.001).

The correlation between BMD and ferritin, Ca, P, PTH, and vitamin 25 OH D levels is shown in Table 3. BMD showed a strong and significant negative correlation with ferritin (r = −0.791, *p* < 0.001), a positive correlation with Ca (r = 0.499, *p* = 0.008), and a weak but significant correlation with PTH (r = −0.441, *p* = 0.021), but no statistically significant correlation with P or vitamin 25 OH D.

The correlation between BMD and body weight (sds), height (sds), and BMI (sds) is shown in Table 4. BMD showed a positive and significant correlation with body weight (sds) and body height (sds) (r = 0.520, *p* = 0.005 and r = 0.580, *p* = 0.002). BMD showed a weak positive correlation with BMI (sds), which was statistically not significant (r = 0.252, *p* = 0.204).

Table 5 evaluates the factors associated with the development of osteoporosis in children with thalassemia major using univariate and multivariate logistic regression analyses. According to our univariate model analysis, body weight (sds) (OR: 0.188, 95% CI: 0.039–0.898, *p*: 0.036) and ferritin (OR: 1.001, 95% CI: 1.000–1.003, *p*: 0.013) were significant. However, this relationship was not significant according to multivariate analysis.

## 4. Discussion

In our study, osteoporosis was diagnosed in 22.2% of children and adolescents with TM, and the frequency of osteoporosis increased with age. We also found that the presence of osteoporosis increased in patients with high ferritin levels, increased splenic size, short height, and low body weight. Moreover, Ca, P, and vitamin 25 OH D levels were lower, while ALP and PTH levels were higher in patients with TM and osteoporosis.

Despite treatment, osteoporosis is observed in 30–50% of thalassemia patients [16,17,18,19,20]. Osteoporosis is an important cause of morbidity in children and adults with TM due to causing bone marrow enlargement; hypogonadism; iron overload; chelator toxicity; Ca, zinc, and vitamin D deficiencies; and inadequate physical activity. All of these can lead to impaired balance of bone remodelling. These factors cause bone loss and osteoporosis by inhibiting osteoblast activation or increasing osteoclast function [21,22,23,24]. In our study, patients with osteoporosis had lower Ca, P, and vitamin 25 OH D levels but higher ALP and PTH levels. In patients with TM, hepatic vitamin D hydroxylation is impaired by iron chelation; therefore, the prevalence of vitamin 25 OH D deficiency is quite high in these patients. Singh et al. reported a prevalence of 80% for vitamin 25 OH D deficiency in patients with thalassemia and found a positive correlation between vitamin D level and lumbar vertebra BMD Z score [23]. However, other studies on patients with TM have not found a significant correlation between vitamin D and BMD [6,18]. In this study, although vitamin D levels were low in both groups, they were lower in the group with osteoporosis. A correlation analysis between BMD and vitamin 25 OH D did not indicate any significant correlation. However, lower serum Ca levels and PTH levels being in the upper limit of normal in the ostoporotic group indicates that they do not have sufficient vitamin D. Hence, other studies have demonstrated a low BMD and reduced serum Ca in patients with TM. This is probably due to reduced intestinal Ca absorption from the intestine secondary to vitamin D deficiency [24,25]. In the treatment of osteoporosis due to thalassemia, pharmacological treatment is important in addition to general measures such as control of anaemia, adequate chelation, regular exercise, and control of comorbidities. For treatment, bone mass can be increased with Ca given during bone development periods, and bone loss and fractures can be prevented with vitamin D to be added to the treatment [23].

The effect of gender differences on the development of osteoporosis in thalassaemia patients is a controversial issue. Although osteoporosis is more common in females in the general population, there are studies reporting that men are more affected among thalassaemia patients. In the study by Alswat et al. which included patients aged 19–25 years and in the study by Kyriakou et al. which evaluated individuals with a mean age of 31.4 years, it was reported that male patients were more frequently and more severely affected by osteoporosis [26,27]. On the other hand, no significant difference was found between genders in the study by Shamshirsaz et al., in which thalassemia major patients aged 10–20 years were evaluated [28]. In our study conducted in pediatric patients, no statistically significant difference was found between the groups in terms of gender distribution. Osteoporosis is likely to be more common in male patients with advancing age.

Many studies have shown a negative correlation between BMD Z scores and patient age. The prevalence of osteoporosis increases with patient age [20,29,30]. Similar to the literature, our study results indicated a higher mean age in patients with osteoporosis.

Serial measurements of serum ferritin continue to be a reliable method and the simplest method of determining iron overload and the efficacy of chelation therapy in patients with TM [31]. Iron overload has a negative impact on bone mineralisation. In children with TM, BMD was significantly affected by ineffective chelation. Since patients with TM are dependent on transfusions, increased ferritin levels cause iron deposition in many organs. Notably, in bones, increased iron accumulation impairs bone mineralization and causes osteoporosis. While several studies in the literature have reported that there is no correlation between serum ferritin level and BMD, several others have reported a negative relationship between serum ferritin level and BMD [32,33,34,35]. Our study results indicated significantly higher ferritin levels in patients with osteoporosis. A strong and significant negative correlation was found between BMD and ferritin.

In TM patients, the diagnosis can be made 6 months after birth, when the HgF level starts to return to adult values. In thalassaemia, iron accumulation occurs in many organs due to intermittent erythrocyte transfusion. The limit for starting iron chelation is after 12–20 transfusions or when ferritin rises above 1000 mg/dL according to the Health Implementation Communiqué (HIC) rules in our country. Again, according to HIC rules, chelation treatment should be discontinued when ferritin falls below 500 mg/dL. Ferritin is preferred because it is a common, inexpensive and easily monitored parameter [36]. In our patients, ferritin level is monitored every 3 months, and the appropriate iron chelator dose is adjusted. In cases where chelator treatment used to reduce iron load is inadequate, BMD decreases and liver vitamin D hydroxylation is impaired in patients receiving high chelator doses. Therefore, providing the appropriate chelator dose is essential for bone health. However, chelators are inevitably used to reduce damage caused by high ferritin levels [37]. In patients with TM who frequently receive transfusions and chelation therapy, the destruction of bones significantly reduces BMD. Studies conducted at different times have shown that patients with osteoporosis had a higher rate of transfusions and a higher chelator dose [17,29,38]. Our study also demonstrated that patients with osteoporosis had a higher transfusion frequency and chelator dose, although the differences did not reach statistical significance. However, patients with osteoporosis had significantly higher ferritin levels. The need for a higher chelator dose may be considered.

The complications of iron overload in children include growth retardation and delayed puberty [39]. Our osteoporotic group had significantly lower body weight and height. A significant positive correlation was found between BMD and body weight and height. One patient in the osteoporotic group was in the prepubertal period, while two others had puberte tarda.

### Study Limitations

Our study has several limitations. This was a retrospective study with a small sample size of 27 children. In addition, as this was a single-center study, it is hard to generalize the findings. Finally, there are no data on lifestyle factors such as dietary habits and physical activity levels. However, our study provides important results regarding the risk of osteoporosis in pediatric TM patients. Multicentre studies including more pediatric patients are needed.

## 5. Conclusions

Osteoporosis is a common complication of TM, the early detection of which is of paramount importance. Therefore, screening BMD measurements routinely after 10 years of age and evaluating other bone parameters regularly at routine intervals in patients with TM are important. Patients with high ferritin levels, increased splenomegaly, short height, and low body weight and patients in the high-risk group with low Ca, P, and vitamin 25 OH D levels and high ALP and PTH levels should be followed up more frequently. We suggest that Ca and vitamin D treatments can be administered to support bone health. Last but not least, a multidisciplinary approach is necessary to maintain bone health for patients with TM. Hematology specialists are responsible for the control of anemia, chelator therapy, and necessary referrals for possible complications, and endocrine specialists should undertake the management of osteoporosis. Physiotherapy for regular exercise and dietitian support for appropriate nutrition programmes should also be provided.

## Figures and Tables

**Table 1:** Comparison of demographic and clinical data of the patients.

Variables	Group 1 (*n* = 6)	Group 2 (*n* = 21)	*p* Value
Gender (Male/Female)	4/2	12/9	0.685
Age (years)	13.83 ± 2.85	7.95 ± 5.05	0.012
Body weight (sds)	−2.18 ± 0.69	−0.77 ± 1.18	0.012
Height (sds)	−2.85 ± 0.75	−0.89 ± 1.36	0.004
Chelator dose (mg/kg)	29.43 ± 12.87	22.90 ± 8.41	0.128
Transfusion frequency(days)	21.50 ± 8.43	23.52 ± 4.75	0.419
Bone densitometry, sds	−2.84 ± 1.42	0.25 ± 1.06	0.001
Splenic size	189.17 ± 6.43	126.48 ± 37.95	0.001

Data are represented as mean ± standart deviation (SD). Abbreviations: Group 1—osteoporosis, Group 2—non-osteoporosis.

**Table 2:** Comparison of biochemical values of the patients.

Variables	Group 1 (*n* = 6)	Group 2 (*n* = 21)	*p* Value
Calcium	8.38 ±0.22	9.47 ± 0.56	<0.001
Phosphorus	4.33 ± 0.56	5.02 ± 0.72	0.038
Alkaline phosphatase	222.17 ± 83.90	188.57 ± 82.50	0.232
Parathyroid hormone	51.83 ±24.11	34.91 ± 17.76	0.075
Vitamin 25 hydroxy D	12.32 ± 7.68	17.82 ± 7.41	0.180
Ferritin	5306 ± 1506	2020 ± 1205	0.001

Data are represented as mean ± standart deviation (SD). Abbreviations: Group 1—osteoporosis, Group 2—non-osteoporosis.

**Table 3:** Correlation between biochemical variables and bone mineral density.

Variables	Ferritin	Calcium	Phosphorus	Parathyroid Hormone	Vitamin 25 Hydroxy D
	*r*; *p*	*r*; *p*	*r*; *p*	*r*; *p*	*r*; *p*
BMD	−0.791; <0.001	0.499; 0.008	0.220; 0.270	−0.441; 0.021	0.222; 0.266

Abbreviations: BMD—bone mineral density.

**Table 4:** Correlation between bone mineral density and body weight, height and body mass index.

Variables	Body Weight	Height	BMI
	*r*; *p*	*r*; *p*	*r*; *p*
BMD	0.520; 0.005	0.580; 0.002	0.252; 0.204

Abbreviations: BMD—bone mineral density; BMI—body mass index.

**Table 5:** Factors associated with the development of osteoporosis among children with thalassemia major according to univariate and multivariate logistic regression analyses.

Parameters	Univariate Model		Multivariate Model	
	OR (Cl 95%)	*p* Value	OR (Cl 95%)	*p* Value
Age	1.182 (0.963–1.450)	0.109		
Gender	1.500 (0.223–10.077)	0.677		
Body weight	0.188 (0.039–0.898)	0.036	0.044 (0.000–11.953)	0.275
Height	0.065 (0.004–1.151)	0.062		
Calcium	0.000 (0.000–1.463)	0.058		
Phosphorus	0.224 (0.047–1.063)	0.060		
Alkaline phosphatase	1.005 (0.994–1.015)	0.385		
Parathyroid hormone	1.039 (0.992–1.089)	0.107		
Vitamin 25 hydroxy D	0.885 (0.753–1.039)	0.135		
Ferritin	1.001 (1.000–1.003)	0.013	1.002 (0.999–1.005)	0.118

## Data Availability

The datasets used and/or analyzed during the current study are available from the corresponding author on reasonable request.

## Abbreviations

The following abbreviations are used in this manuscript:

TM	Thalassemia major
BMD	Bone mineral density
BMI	Body mass index

## References

[1] Yapıcı G., Kurt Ö., Öner S., Şaşmaz T., Buğdaycı R., Tamam A. (2009). Kronik kan transfüzyonu alan beta-talasemi major ve orak hücreli anemi hastalarında görülen komplikasyonlar. CMJ.

[2] Canatan D. (2011). Haemoglobinopathy Prevention Program in Turkey. Thalass. Rep..

[3] Gulati R., Bhatia V., Agarwal S.S. (2000). Early onset of endocrine abnormalities in beta-thalassemia major in a developing country. J. Pediatr. Endocrinol. Metab..

[4] Mahmoud R.A., Khodeary A., Farhan M.S. (2021). Detection of endocrine disorders in young children with multi-transfused thalassemia major. Ital. J. Pediatr..

[5] Daar S., Al-Naamani K., De Sanctis V., Al Rahbi S., Al Zadjali S., Khan H., Panjwani V., Al-Khabori M. (2023). Mortality and complications in Omani patients with beta-thalassemia major: A long-term follow-up study. Acta Biomed..

[6] Morabito N., Lasco A., Gaudio A., Crisafulli A., Di Pietro C., Meo A., Frisina N. (2002). Bisphosphonates in the treatment of thalassemia-induced osteoporosis. Osteoporos. Int..

[7] Mamtani M., Kulkarni H. (2010). Bone recovery after zoledronate therapy in thalassemia-induced osteoporosis: A meta-analysis and systematic review. Osteoporos. Int..

[8] Trotta A., Corrado A., Cantatore F.P. (2010). La terapia anabolica nella osteoporosi indotta da beta-talassemia major: Caso clinico e revisione della letteratura [Anabolic therapy of induced osteoporosis in beta-thalassaemia major: Case report and literature review]. Reumatismo.

[9] Gaudio A., Morabito N., Xourafa A., Macrì I., Meo A., Morgante S., Trifiletti A., Lasco A., Frisina N. (2008). Bisphosphonates in the treatment of thalassemia-associated osteoporosis. J. Endocrinol. Investig..

[10] Baim S., Binkley N., Bilezikian J.P., Kendler D.L., Hans D.B., Lewiecki E.M., Silverman S. (2008). Official Positions of the International Society for Clinical Densitometry and executive summary of the 2007 ISCD Position Development Conference. J. Clin. Densitom..

[11] Toumba M., Skordis N. (2010). Osteoporosis syndrome in thalassaemia major: An overview. J. Osteoporos..

[12] Kröger H., Kotaniemi A., Kröger L., Alhava E. (1993). Development of bone mass and bone density of the spine and femoral neck--a prospective study of 65 children and adolescents. Bone Miner..

[13] Nayir Buyuksahin H., Dogru D., Gözmen O., Ozon A., Portakal O., Emiralioglu N., Haliloglu M., Kılıc K., Vardar Yaglı N., Yıldırım D. (2023). Cystic fibrosis related bone disease in children: Can it be predicted?. Clin. Nutr..

[14] Lasco A., Morabito N., Gaudio A., Buemi M., Wasniewska M., Frisina N. (2001). Effects of hormonal replacement therapy on bone metabolism in young adults with beta-thalassemia major. Osteoporos. Int..

[15] Turkish Pediatric Endocrinology and Diabetes Society Bone Mineral Density. https://www.ceddcozum.com/BoneMineralDensity.

[16] Perifanis V., Vyzantiadis T., Tziomalos K., Vakalopoulou S., Garipidou V., Athanassiou-Metaxa M., Harsoulis F. (2007). Effect of zoledronic acid on markers of bone turnover and mineral density in osteoporotic patients with betathalassaemia. Ann. Hematol..

[17] Baytan B., Sağlam H., Erdöl Ş., Beyazıt A.N., Özgür T., Güneş A.M., Günay Ü. (2008). Talasemi majorlu vakalarda endokrin komplikasyonların değerlendirilmesi. J. Curr. Pediatr..

[18] Chow L.C., Lee B.S., Tang S.O., Loh E.W., Ng S.C., Tan X.Y., Ahmad Noordin M.N., Ong G.B., Chew L.C. (2024). Iron burden and endocrine complications in transfusion-dependent thalassemia patients In Sarawak, Malaysia: A retrospective study. Med. J. Malays..

[19] Chahkandi T., Norouziasl S., Farzad M., Ghanad F. (2017). Endocrine disorders in beta thalassemia major patients. Int. J. Pediatr..

[20] Bielinski B.K., Darbyshire P., Mathers L., Boivin C.M., Shaw N.J. (2001). Bone density in the Asian thalassaemic population: A cross-sectional review. Acta Paediatr..

[21] Uğur S., Kurtoğlu E. (2022). Talasemide kemik metabolizması bozuklukları, osteoporoz ve tedavisi. Talasemi.

[22] Mahachoklertwattana P., Chuansumrit A., Sirisriro R., Choubtum L., Sriphrapradang A., Rajatanavin R. (2003). Bone mineral density, biochemical and hormonal profiles in suboptimally treated children and adolescents with beta-thalassaemia disease. Clin. Endocrinol..

[23] Singh K., Kumar R., Shukla A., Phadke S.R., Agarwal S. (2012). Status of 25-hydroxyvitamin D deficiency and effect of vitamin D receptor gene polymorphisms on bone mineral density in thalassemia patients of north ındia. Hematology.

[24] Goyal M., Abrol P., Lal H. (2010). Parathyroid and calcium status in patients with thalassemia. IJCB.

[25] Soliman A., Adel A., Bedair E., Wagdy M. (2008). An adolescent boy with thalassemia major presenting with bone pain, numbness, tetanic contractions and growth and pubertal delay: Panhypopituitarism and combined vitamin D and parathyroid defects. Pediatr. Endocrinol. Rev..

[26] Alswat K.A. (2017). Gender Disparities in Osteoporosis. J. Clin. Med. Res..

[27] Kyriakou A., Savva S.C., Savvides I., Pangalou E., Ioannou Y.S., Christou S., Skordis N. (2008). Gender differences in the prevalence and severity of bone disease in thalassaemia. Pediatr. Endocrinol. Rev..

[28] Shamshirsaz A.A., Bekheirnia M.R., Kamgar M., Pakbaz Z., Tabatabaie S.M., Bouzari N., Pourzahedgilani N., Azarkeivan A., Hashemi S.R., Moosavi F. (2007). Bone mineral density in Iranian adolescents and young adults with thalassemia major. Pediatr. Hematol. Oncol..

[29] Pirinççioğlu A.G., Akpolat V., Köksal O., Haspolat K., Söker M. (2011). Bone mineral density in children with beta-thalassemia major in Diyarbakir. Bone.

[30] Terpos E., Voskaridou E. (2010). Treatment options for thalassemia patients with osteoporosis. Ann. N. Y. Acad. Sci..

[31] Angelucci E., Brittenham G.M., McLaren C.E., Ripalti M., Baronciani D., Giardini C., Galimberti M., Polchi P., Lucarelli G. (2000). Hepatic iron concentration and total body iron stores in thalassemia major. N. Engl. J. Med..

[32] Pollak R.D., Rachmilewitz E., Blumenfeld A., Idelson M., Goldfarb A.W. (2000). Bone mineral metabolism in adults withb-thalassaemia major and intermedia. Br. J. Haematol..

[33] Inati A., Noureldine M.A., Mansour A., Abbas H.A. (2015). Endocrineand bone complications in β- thalassemia intermedia: Current understanding and treatment. Biomed. Res. Int..

[34] Eren E., Yilmaz N. (2005). Biochemical markers of bone turnover and bone mineral density in patients with beta-thalassaemia major. Int. J. Clin. Pract..

[35] Sadat-Ali M., Sultan O., Al-Turki H., Alelq A. (2011). Does high serum iron level induce low bone mass in sickle cell anemia?. Biometals.

[36] Aydinok Y., Bayraktaroglu S., Yildiz D., Alper H. (2011). Myocardial iron loading in patients with thalassemia major in Turkey and the potential role of splenectomy in myocardial siderosis. J. Pediatr. Hematol. Oncol..

[37] Fernandes J.L., Loggetto S.R., Veríssimo M.P., Fertrin K.Y., Baldanzi G.R., Fioravante L.A., Tan D.M., Higa T., Mashima D.A., Piga A. (2016). A randomized trial of amlodipine in addition to standard chelation therapy in patients with thalassemia major. Blood.

[38] Haidar R., Musallam K.M., Taher A.T. (2011). Bone disease and skeletal complications in patients with β thalassemia major. Bone.

[39] Borgna-Pignatti C., Galanello R. (2004). Thalassemias and related disorders: Quantitative disorders of hemoglobin synthesis. Wintrobe’s Clinical Haematology.

